# Metabolomic diferences between COVID-19 and H1N1 influenza induced ARDS

**DOI:** 10.1186/s13054-021-03810-3

**Published:** 2021-11-15

**Authors:** Jose Angel Lorente, Nicolas Nin, Palmira Villa, Dovami Vasco, Ana B. Miguel-Coello, Ignacio Rodriguez, Raquel Herrero, Oscar Peñuelas, Jesús Ruiz-Cabello, Jose L. Izquierdo-Garcia

**Affiliations:** 1 CIBER de Enfermedades Respiratorias, CIBERES, Instituto de Salud Carlos III, Madrid, Spain; 2 Department of Critical Care, Hospital Universitario de Getafe, Madrid, Spain; 3 Universidad Europea de Madrid, Madrid, Spain; 4 Hospital Español, Montevideo, Uruguay; 5 Centro de Asistencia a La Investigación Bioimagen Complutense, Universidad Complutense de Madrid, Madrid, Spain; 6 Center for Cooperative Research in Biomaterials (CIC biomaGUNE), Basque Research and Technology Alliance (BRTA), Paseo de Miramon 182, 20014 Donostia San Sebastián, Spain; 7 Instituto Pluridisciplinar, Universidad Complutense de Madrid, Paseo Juan XXIII, 1, Madrid, Spain; 8 Departamento de Química en CC. Farmacéuticas, Facultad de Farmacia, Universidad Complutense de Madrid, Madrid, Spain

**Keywords:** ARDS, COVID-19, H1N1 influenza, Metabolomics, NMR, Diagnosis

## Abstract

**Background:**

Acute respiratory distress syndrome (ARDS) is a type of respiratory failure characterized by lung inflammation and pulmonary edema. Coronavirus disease 2019 (COVID-19) is associated with ARDS in the more severe cases. This study aimed to compare the specificity of the metabolic alterations induced by COVID-19 or Influenza A pneumonia (IAP) in ARDS.

**Methods:**

Eighteen patients with ARDS due to COVID-19 and twenty patients with ARDS due to IAP, admitted to the intensive care unit. ARDS was defined as in the American-European Consensus Conference. As compared with patients with COVID-19, patients with IAP were younger and received more often noradrenaline to maintain a mean arterial pressure > 65 mm Hg. Serum samples were analyzed by Nuclear Magnetic Resonance Spectroscopy. Multivariate Statistical Analyses were used to identify metabolic differences between groups. Metabolic pathway analysis was performed to identify the most relevant pathways involved in ARDS development.

**Results:**

ARDS due to COVID-19 or to IAP induces a different regulation of amino acids metabolism, lipid metabolism, glycolysis, and anaplerotic metabolism. COVID‐19 causes a significant energy supply deficit that induces supplementary energy-generating pathways. In contrast, IAP patients suffer more marked inflammatory and oxidative stress responses. The classificatory model discriminated against the cause of pneumonia with a success rate of 100%.

**Conclusions:**

Our findings support the concept that ARDS is associated with a characteristic metabolomic profile that may discriminate patients with ARDS of different etiologies, being a potential biomarker for the diagnosis, prognosis, and management of this condition.

**Graphical Abstract:**

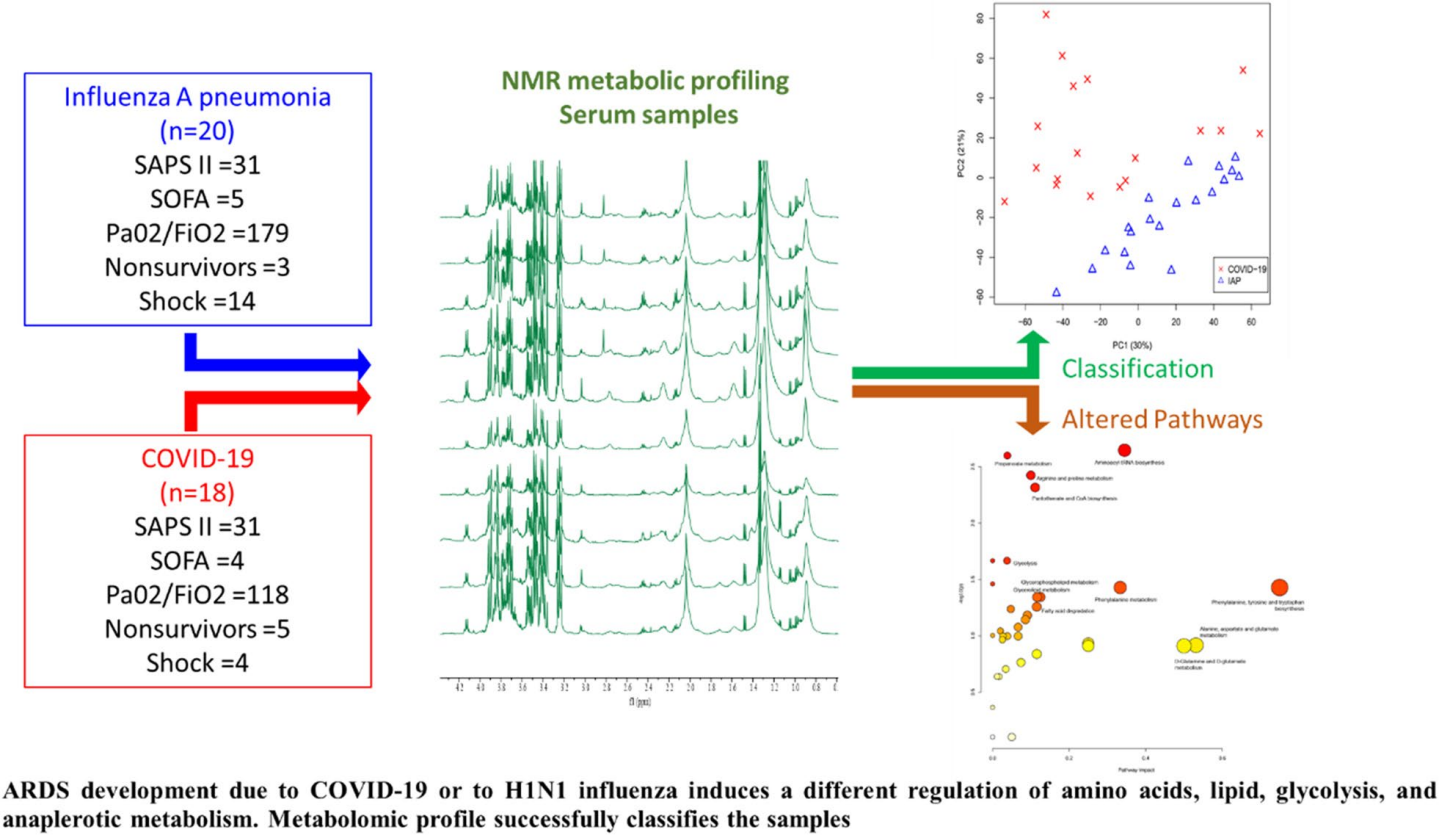

**Supplementary Information:**

The online version contains supplementary material available at 10.1186/s13054-021-03810-3.

## Background

The current Coronavirus disease 2019 (COVID-19) pandemic, caused by the Severe Acute Respiratory Syndrome Coronavirus 2 (SARS-CoV-2) [1], strained critical care resources in many countries, and the management of lung injury in these patients posed a tremendous challenge for clinicians [2]. Several patients with COVID-19 developed severe acute respiratory distress syndrome (ARDS) with mortality rates around 30% [3] in the first pandemic wave. ARDS is characterized by lung inflammation and hyperpermeability pulmonary edema. Currently, the diagnosis of ARDS is based on the presence of clinical, physiological, and radiological criteria [4–6]. Unlike other clinical conditions, to date, there are no specific molecular markers that help in the prognosis of this condition. Advancement in the understanding of the pathogenesis of ARDS is necessary for designing innovative and effective therapeutic approaches.

Molecular approaches are needed to understand the mechanisms of ARDS induced by COVID-19. Of particular interest is the use of metabolomics for the characterization of this condition. The metabolome reflects early and specific alterations in the pathophysiological state of biological systems. In this context, Magnetic Resonance Spectroscopy (MRS) emerges as a highly potential tool for studying metabolic disorders in respiratory diseases [7]. Several studies have proved the potential of MRS-based metabolomics to monitor patients with ARDS induced by respiratory infections [8–10]. A previous study compared the specific metabolic fingerprint of ARDS patients with either influenza A pneumonia (IAP) caused by the H1N1-2009 influenza virus or pneumonia caused by *Streptococcus pneumoniae* [11]. Here, we used a similar approach for the characterization of the metabolic fingerprint of COVID-19-induced ARDS. We compared COVID-19 and IAP patients to identify the metabolic reprogramming involved in these two conditions. The identification of metabolic pathways involved in ARDS caused by the H1N1-2009 influenza virus or by SARS-CoV-2 will improve our understanding of the pathogenesis of COVID-19. Finally, as a proof of concept of the diagnostic potential of these metabolic biomarkers, we developed a predictive model to identify the etiological pathogens responsible for ARDS.

## Methods

### Experimental design

Patients with Coronavirus disease 2019 (COVID-19, *n* = 18) were recruited from March 1, 2020, to June 31, 2020, in Hospital Universitario de Getafe, Madrid. Metabolic profile from ARDS patients with H1N1-2009 influenza pneumonia was acquired and analyzed in our previous study [12]. Serum samples from ARDS patients with H1N1-2009 influenza virus pneumonia were obtained in Hospital Universitario de Getafe, Madrid, Spain (*n* = 10) and Hospital del Mar, Barcelona, Spain (*n* = 10) during the 2009 pandemic and stored at − 80 °C until NMR analysis in September of 2017. H1N1-2009 infection was confirmed by RT-PCR or either nasopharyngeal swab samples or tracheal secretions. In all cases, serum samples were obtained within 24 h of presentation to the emergency department. Blood samples were collected in BD Vacutainer tubes after each participant signed informed consent. After collection, the sample was left at room temperature for 30 min to clot. The clot was removed by centrifugation at 1500 × g for 10 min at 4 °C. The resulting supernatant was immediately transferred into 2 ml Eppendorf tubes and stored at − 80 °C.

Inclusion criteria for both studies were: age ≥ 18 years, diagnosis of ARDS, confirmed infection by SARS-CoV-2 or H1N1-2009 influenza virus by real-time reverse transcription-polymerase chain reaction (RT-PCR) of nasopharyngeal swab samples, and admission to the Intensive Care Unit (ICU). ARDS was defined as in the American-European Consensus Conference (AECC) [13]. All patients were mechanically ventilated.

Clinical information was obtained by retrospective chart review, and data of the Sequential Organ Failure Assessment (SOFA) and the Simplified Acute Physiologic Score-II (SAPS II) scores on admission, the presence of renal or cardiovascular failure (SOFA score of the respective component > 2) [14] and status at hospital discharge (hospital mortality) were collected.

### NMR data acquisition

Serum samples were collected after each participant signed informed consent within 24 h of ICU admission and examined (40 µl of serum) by high-resolution magic angle spinning (HR-MAS) NMR operating at 4 °C to reduce metabolic degradation. HR-MAS NMR was performed at 500.13 MHz using a Bruker AMX500 spectrometer 11.7 T. HR-MAS NMR has several strengths for clinical studies [15]; (1) signals in NMR spectrum have the same sensitivity independently of the properties of the metabolite; (2) a combination of NMR techniques enables the unambiguous identification of metabolic signals; (3) HR-MAS NMR enables analysis of intact samples, which is essential considering that factors associated with sample preparation contribute to analytical variability; (4) metabolite profiles obtained by NMR are virtually independent of the operator and instrument used, which provides a high degree of reliability to the derived results.

Samples were placed into a 50 μl zirconium oxide rotor using a rinsed with a cylindrical insert, together with 15 µl of 0.1 mM solution of TSP in deuterium water (D_2_O), and spun at 4000 Hz spinning rate to remove the effects of spinning sidebands from the acquired spectra. Several bidimensional homonuclear and heteronuclear experiments such as standard gradient-enhanced correlation spectroscopy (COSY), ^1^H–^1^H total correlated spectroscopy (TOCSY), and gradient-selected heteronuclear single quantum correlation (HSQC) protocols were performed to carry out component assignments. Between consecutive two-dimensional (2D) spectra, a control ^1^H NMR spectrum was continuously measured to detect metabolic degradation or microbiologically contamination. No metabolic differences were noted in the signals of multiple spectra acquired under the same conditions. Standard solvent-suppressed spectra were grouped into 32,000 data points, averaged over 256 acquisitions [16]. The data acquisition lasted 13 min using a sequence based on the first increment of the nuclear Overhauser effect spectroscopy (NOESY) pulse sequence to effect suppression of the water. Sample acquisitions were performed using a spectral width of 8333.33 Hz before Fourier transformation, and the free induction decay (FID) signals were multiplied by an exponential weight function corresponding to a line broadening of 0.3 Hz. Spectra were referenced to the TSP singlet at 0 ppm chemical shift.

NMR data were processed for statistical analysis and metabolic quantification. The chemical shifts region from 5.00 to 5.20 ppm was excluded from the analysis to remove the random and known effects of variation in the water resonance suppression. Similarly, the chemical shifts region from 0 to 0.04 ppm containing the internal reference (TSP) was excluded from the statistical analysis. Phase and baseline correction (Whittaker method) were performed automatically using MestRenova v. 8.1 software (Mestrelab Research S.L., Santiago de Compostela, Spain). For statistical analysis, ^1^H NMR spectra were automatically data-reduced to integral segments or buckets of equal length (*δ* = 0.01 ppm) to compensate for variations in resonance positions [17], and they were normalized to the total sum of the spectral regions. For metabolic quantification, full resolution spectra were normalized to the total sum of the spectral regions too. Relative intensity was calculated as the initial intensity normalized to the total sum of the spectral regions.

### Statistical analysis

Quantitative and qualitative variables were compared by the Student´s t-test or the Chi-square test, respectively. A *p* value less than 0.05 was considered statistically significant. The statistical package SPSS IBM Statistics 19.0 was used for the analysis. Descriptive data are presented as mean (SD) for continuous variables and percentages for discrete variables.

Principal Components Analysis (PCA) [18] was performed over binned NMR spectral data using the Metabonomic package (rel.3.3.1) [19] to analyze in ARDS patients the differences between SARS-CoV-2 and H1N1-2009 influenza infection. In PCA, the data collected on a set of samples are resolved into principal components. The first principal component is defined by the spectral profile (loadings) in the data that describes most of the variation; the second principal component, orthogonal to the first, is the second-best profile describing the variation, and so on. The principal components are composed of so-called scores and loadings. Loadings contain information about the variables (chemical shifts) in the dataset, and scores hold information on samples (intensities) in the dataset. Before PCA, NMR processed data were centered, and Pareto scaled [20]. Hotteling’s T2 test [21] identified the NMR areas (NMR bins) from the PCA loading matrix responsible for group clustering. The NMR signals in the identified NMR areas were individually integrated into full resolution spectra for metabolic quantification using the Global Spectral Deconvolution algorithm of MestRenova v. 8.1 (Mestrelab Research S.L., Santiago de Compostela, Spain). Metabolites identification was performed manually using Chenomx Profiler tool [22]. Metabolites assignments were confirmed by analyzing 2D-NMR spectra using MestRenova software and the Human Metabolome Database [23].

For the metabolic quantification, statistical significance was determined using a Bonferroni corrected Student’s t-test [24], assuming unequal variance with *p* < 0.05 considered significant.

Partial Least Square Discriminant Analysis (PLS-DA) [25] was developed as a classificatory model using MetaboAnalyst v.5.0 [26] to differentiate in ARDS patients those due to IAP from those due to COVID-19. PLS-DA models have commonly used classification methods for analyzing high-dimensional data. The number of latent variables used to develop the PLS-DA model were evaluated by R2 and Q2 robustness parameters. R2 can be considered a metric of how the algorithm fits the training data, and Q2 is a metric of algorithm performance on test data [27]. Q2 parameter, which evaluates the classification functions derived from the probability of belonging to each group, was computed by leave-one-out cross-validation (LOOCV) to minimize the variance in training [28]. Three PLS components were selected to develop a classificatory model based on the best robustness results (R2 = 0.94; Q2 = 0.89). Model performance was evaluated by Prediction accuracy during cross-validation and the Area Under de Curve Receiver Operating Characteristic (AUC-ROC). AUC-ROC curve is a performance measurement for classification problems at various threshold settings. The ROC curve is plotted with True Positive Rate (sensitivity) against the False Positive Rate (1-specificity).

### Metabolic pathways analyses

Metabolic pathway analysis was performed using The Pathway Analysis module [29] of Metaboanalyst v.5.0 [26] that combines results from robust pathway enrichment analysis [30] with pathway topology analysis [31] to help researchers identify the most relevant pathways involved in the conditions under study.

Briefly, pathway enrichment analysis examines whether metabolites in predefined pathways are at the top or bottom of a ranked list. In contrast, pathway topology analysis applies graph theory to measure the importance of an experimentally identified metabolite in a predefined metabolic pathway. KEGG metabolic pathways were used as the backend knowledgebase, the selected pathway enrichment analysis method was GlobalAncova [32], node importance measure for topological analysis was out-degree centrality. Centrality is a standard metric used in graph theory to estimate the relative importance of individual nodes to the overall network [33]. Out-degree is the number of outgoing links or the number of successor nodes.

## Results

### Characteristics of study patients and laboratory findings on admission

We compared ARDS patients with COVID-19 (*n* = 18) with ARDS patients with IAP (*n* = 20). Compared to patients with COVID-19, patients with IAP were younger and received more often noradrenaline to maintain a mean arterial pressure > 65 mm Hg (Table 1). Disease severity as measured by SAPS II score, SOFA score ad mortality was similar in both groups.

**Table 1:** Clinical characteristics of the study groups

Clinical characteristic	IAP (*n* = 20)	COVID-19 (*n* = 18)	*p* value*
Age	49 (38–71)	60 (54–72)	0.035
Sex (female)	8 (40)	5 (28)	0.506
SAPS II	31 (23–39)	31 (27–34)	0.784
SOFA score	5 (4–7)	4 (3–8)	0.621
PaO_2_/FiO_2_	179 (73–230)	118 (99–219)	0.759
Nonsurvivors	3 (15)	5 (28)	0.438
AKI	8 (45)	9 (45)	1.000
Patients receiving noradenaline	14 (70)	4 (22)	0.004

Values are medians (percentile 25th–percentile 75th), or n and percentage

SAPS II, Simplified Acute Physiology Score II; SOFA score, Sequential Organ Failure Assessment score; AKI, acute kidney injury

*Chi-square test (or Fisher's exact for cells counts < 5) for qualitative variables or Mann Whitney U test for quantitative variables

### Metabolomic analysis

**Fig. 1:**
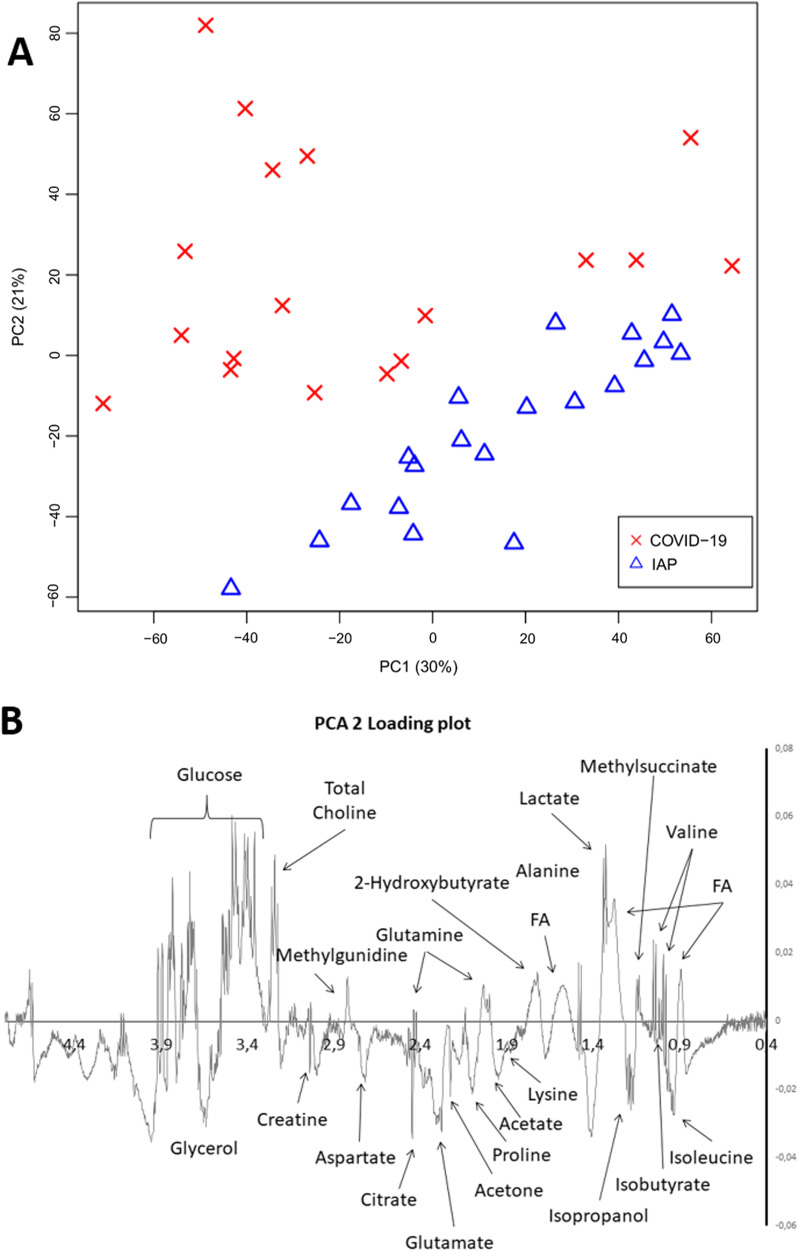
Principal component analysis (PCA) performed on the NMR data of serum samples from ARDS patients diagnosed with Coronavirus disease 2019 (COVID-19) and H1N1-2009 influenza pneumonia (IAP). **A** Score plot discriminated between groups along PC2. **B** PCA 2 loading plot identified the resonances that induce the clustering between IAP and COVID-19 groups. The figure highlights the significant metabolites

**Fig. 2:**
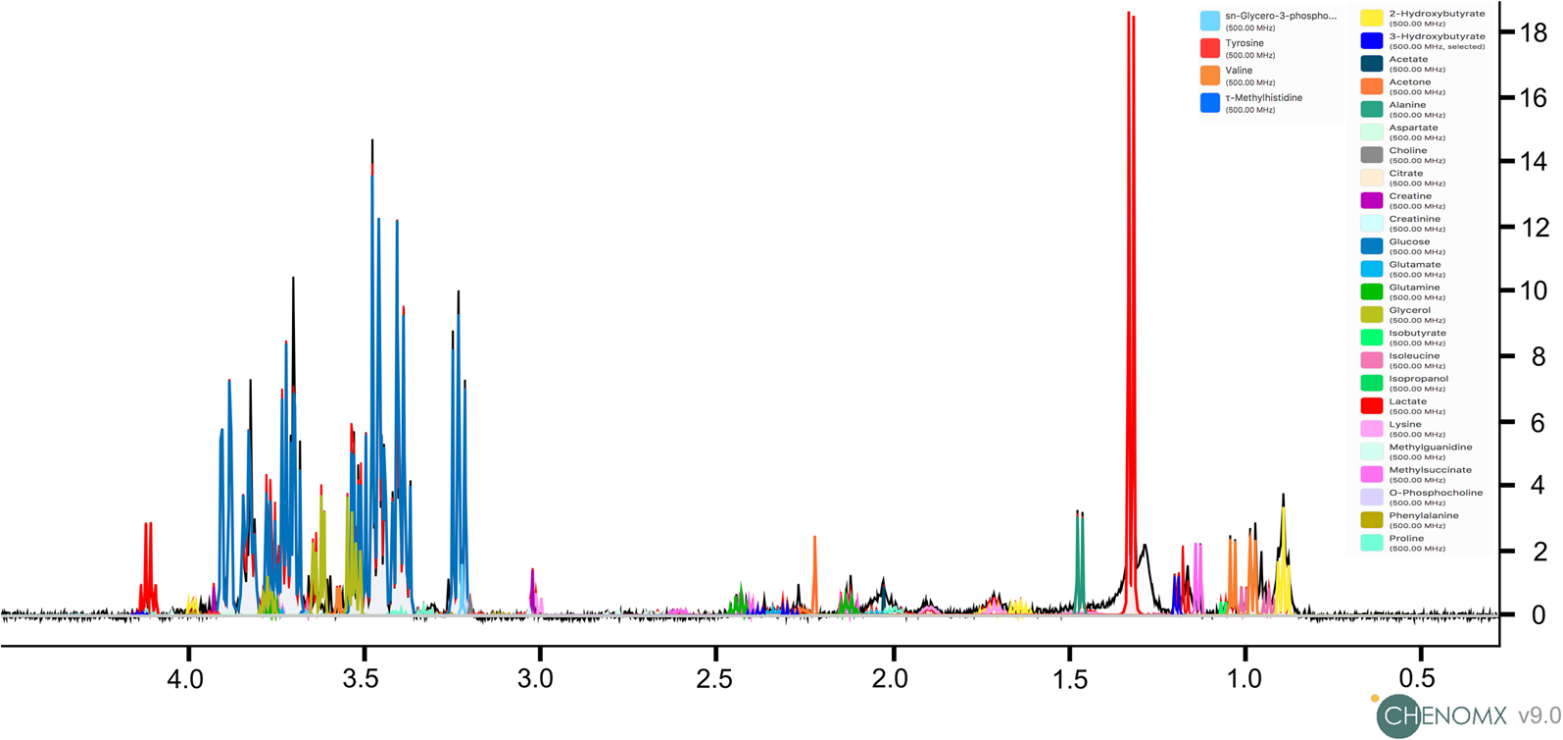
Representative ^1^H HR-MAS NMR spectrum serum sample from COVID-19 ARDS patient. The figure highlights metabolic assignments

An unsupervised classification study with PCA was carried out to analyze in ARDS patients the differences between SARS-CoV-2 and H1N1-2009 influenza infection. PCA (Fig. 1A) provided nearly perfect discrimination between the two groups of subjects. The resonances identified as significantly different by PCA loadings analysis (Fig. 1B) were individually integrated for metabolic quantification. The resonances were identified according to Chenomx Profiler database (Fig. 2) and characteristic cross-peaks from 2D spectra to help in the unequivocal assignation of these metabolites. ARDS patients induced by COVID-19 showed higher metabolic concentrations of free fatty acids, acetone, creatinine, and lactate, and lower metabolic concentrations of valine, 2-hydroxybutyrate, proline, methyl-guanidine, glucose, and tyrosine (Table 2; Additional file 1: Figure S1).

**Table 2:** Relative change in the concentration of the identified metabolites

Metabolite	IAP	COVID-19	*P* value	Relative change (%)
Fatty acids	73.57	85.22	*0.0375*	16
Isoleucine	3.41	2.88	*0.1477*	− 15
Valine	4.46	3.59	*0.0237*	− 19
Isobutyrate	0.09	0.09	*0.9932*	0
Methylsuccinate	0.14	0.12	*0.6781*	− 15
Alanine	5.60	5.29	*0.6368*	− 6
2-Hydroxybutyrate	4.50	2.31	*0.0003*	− 49
Lysine	1.44	1.65	*0.7029*	14
Acetate	1.81	1.93	*0.8101*	7
Proline	74.87	64.23	*0.0049*	− 14
Acetone	2.78	4.02	*0.0424*	45
Glutamate	4.66	7.15	*0.0807*	53
Glutamine	6.81	6.17	*0.6102*	− 9
Citrate	− 0.18	0.04	*0.1807*	− 123
Aspartate	0.43	0.19	*0.0597*	− 56
Methylguanidine	2.45	0.56	*0.0041*	− 77
Creatine	0.88	0.91	*0.8831*	3
Creatinine	0.76	0.57	*0.0358*	− 25
Choline	0.96	1.04	*0.5691*	9
Phosphocholine	1.27	1.42	*0.2644*	12
Glycerophosphocholine	2.61	2.72	*0.6764*	4
Glycerol	7.69	8.72	*0.2456*	13
Creatinine	0.45	1.39	*0.0144*	211
Lactate	8.80	11.10	*0.0257*	26
Glucose	5.86	9.42	*0.0005*	61
Tyrosine	0.34	0.12	*0.0051*	− 66
Phenylalanine	1.24	0.60	0.0619	− 52

IAP, influenza A pneumonia

Statistical significance was determined using a Bonferroni corrected Student’s *t *test assuming significant unequal corrected variance with *p* < 0.05

**Fig. 3:**
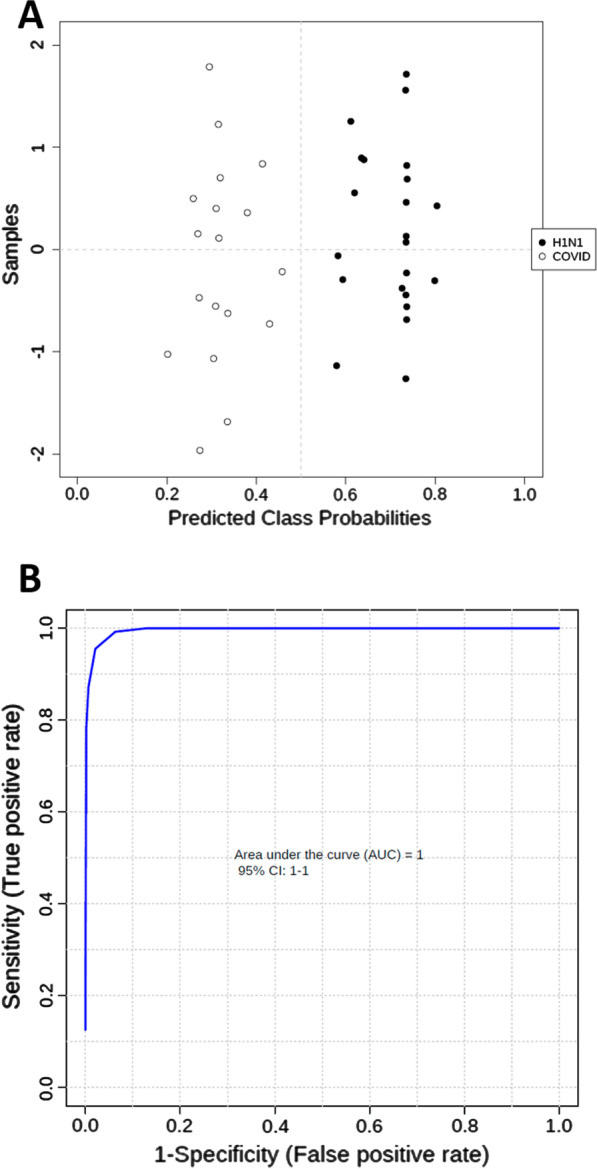
**A** Predicted class probabilities plot (average of the cross-validation) for each sample using the best classifier (based on R2 = 0.94 and Q2 = 0.89 PLS robustness parameters) of the PLS model (3 PLS components). As a balanced subsampling approach is used for model training, the classification boundary is always at the center (*x* = 0.5, the dotted line). **B** Area under de curve receiver operating characteristic (AUCROC) of PLS-DA. COVID, COVID-19; H1N1, influenza A pneumonia

The specific metabolic fingerprint (unassigned ^1^H NMR spectra) was then used to develop a partial least squares discriminant analysis (PLS-DA) to identify SARS-CoV-2 infection. PLS-DA successfully discriminated COVID-19 from IAP (Fig. 3) (Prediction accuracy during cross validation = 100%; AUCROC = 1).

**Fig. 4:**
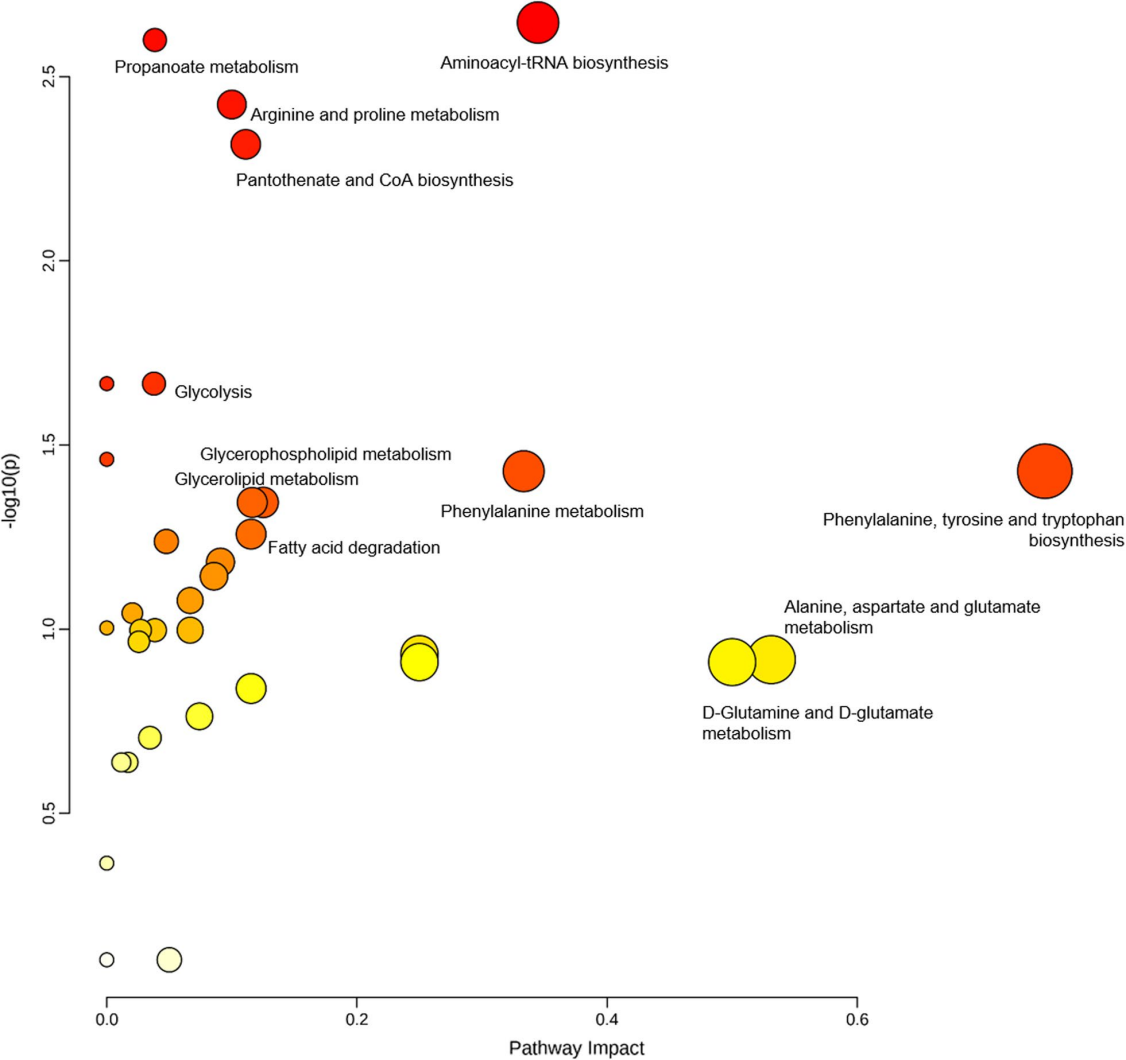
Summary of metabolic pathway analysis. *Y* axis represents the statistical p values from enrichment analysis, and the *X* axis represents the pathway impact value calculated from pathway topology analysis

Metabolic pathway analysis was performed to identify the most relevant pathways involved in ARDS development (Fig. 4; Additional file 1: Table S1). This pathway analysis identified alterations in amino acids biosynthesis and metabolism, glycerolipid metabolism and fatty acid degradation, glycolysis, and anaplerotic metabolism.

## Discussion

The description of the metabolic alterations induced by SARS-CoV-2 infection in ICU patients is fundamental for a better understanding of the pathobiology of the disease. In the present study, we compared the metabolomic profile of ARDS due either to IAP or to COVID-19 by MRS using untargeted multivariate statistical analysis and metabolic pathway analysis. We found that the activation of many metabolic pathways was different between ARDS patients with COVID-19 or IAP. Furthermore, the serum metabolite profile of patients with ARDS discriminates the specific virus infection (H1N1-2009 influenza pneumonia versus SARS-CoV-2 pneumonia). PLS-DA model provided a classification accuracy of 100%. These findings are helpful for the understanding of the pathogenesis of severe COVID-19. Specifically, the metabolomic profile of ARDS in these patients suggests alterations in energy pathways, inflammatory response, and oxidative stress.

Previous studies that have analyzed the metabolism of patients with COVID-19 [34–39] were designed to compare the metabolic profile of COVID-19 patients with healthy controls or to evaluate the metabolic differences between patients with a positive or negative outcome. Thus they cannot discern between the metabolic dysregulation due to SARS-CoV-2 infection or due to ARDS development. To the best of our knowledge, this is the first study designed to compare the metabolic profile of ICU patients with similar severity of ARDS due to COVID-19 or to other viral respiratory infections, e.g. IAP.

We have found specific metabolic differences between ARDS patients induced by COVID-19 or IAP. Most of these metabolic alterations have been previously reported as biomarkers of ARDS or ARDS severity [8–10]. For example, a similar serum metabolic profile including proline, glutamate, phenylalanine, and valine was reported as a sensitive biomarker of ARDS severity (mild, moderate, and severe) [40]. However, we have to consider that, unlike previous studies, in which metabolic changes induced by the viral infection itself or by the occurrence of ARDS cannot be distinguished, IAP and COVID-19 patients in the present study all met the criteria for the diagnosis of ARDS, and disease severity was similar. Thus differences in the metabolic profile herein reported are better explained by virus-specific pathogenetic mechanisms rather than by the occurrence of ARDS or by disease severity.

The ability of patients to normalize energy metabolism has been reported as one of the critical factors determining the COVID-19 progression [39]. Compared with IAP patients, COVID-19 patients showed up-regulation of energy-generating pathways, i.e. glycolysis, fatty acid degradation, CoA biosynthesis, glycerolipids, and glycerophospholipids metabolism. The increase of lactate-to-glucose ratio found in COVID-19 patients is a biomarker of the up-regulation in the glycolysis pathway [41]. In the same context, dysregulation of the choline metabolism and elevated levels of free poly-unsaturated fatty acids are biomarkers of the energy deficiency reported in COVID-19 patients [42]. However, other authors [34, 43] have associated the dysregulation of lipid metabolism with a higher atherosclerotic risk in COVID-19 patients. In the same line, COVID-19 patients showed a higher phenylalanine-tyrosine ratio that has been associated with an adverse outcome [39] and may indicate a higher cardiovascular risk [44] in COVID-19 patients [45]. Other supplementary energy-generating pathways were also up-regulated. The excess of ketone bodies such as acetone suggests that they are used as an alternative energy source. Ketosis could be explained in the context of acute illness and lack of adequate caloric intake.

Alteration in amino acidic metabolism has been reported as one of the key features of ARDS development [8, 9], and it has also been found significantly up-regulated in COVID-19 patients [34, 39, 46, 47]. However, when we compared the metabolic profile of patients with ARDS due to COVID-19 or to IAP, we found that the amino acidic metabolism was decreased in COVID-19 patients. The serum concentrations of branched-chain amino acids (BCAAs), including isoleucine and valine, were decreased in COVID-19 compared with IAP patients. As elevated circulating BCAAs may promote oxidative stress [48], the lower levels of BCAAs in patients with COVID-19 may result in less intense inflammatory response as compared to patients with influenza A [49]. Downregulated BCAAs may also be considered a potential marker of the infection and its further involvement in the dysregulation of pantothenate and CoA biosynthesis [47], as confirmed by the enrichment analysis. Pantothenic acid (vitamin B5) is required for coenzyme A formation and is also essential for α-ketoglutarate and pyruvate dehydrogenase complexes as well as fatty acid oxidation, compromising the mitochondrial energy metabolism [50]. The increase in 2-hydroxybutyric acid, a readout of hepatic glutathione synthesis and marker of oxidative stress [50], and essential amino acids such as proline [51] confirmed more marked inflammatory and oxidative stress responses [52] in IAP than in COVID-19 patients. A previous study also identified dysregulation of propanoate metabolism as a novel pathway in the progression of COVID-19 [46], suggesting potential roles played by gut microbiota in the immune response [53]. Finally, the methyl-guanidine-to-creatinine ratio is an index of hydroxyl radical formation in the lung, and it was identified previously as a specific metabolic pattern of IAP [11].

Several limitations of the study should be acknowledged. First, the metabolic concentrations reported are relative to the total metabolic concentration, and baseline clinical differences among groups should be taken into consideration when interpreting the results. We did not perform absolute quantification because of limitations in sample manipulation for HR-MAS NMR analysis. External validation is required before the application of the specific metabolic fingerprint in clinical practice. Second, despite the overall similarity in disease severity (as measured by the SAPS II score, the SOFA score, and the mortality), patients differed in some characteristics, such as age and the requirement of noradrenaline, that could have an impact on the metabolic profile. Also, oxygenation impairment differed in the two groups, although the difference did not reach statistical significance. Third, some aspects of patients management could differ in the cohorts, as they span some years apart. Specifically, the way patients were mechanically ventilated could have had an impact on the metabolic profile. After the publication of the ARMA trial in 2000 [54], different studies reported changes in the way mechanical ventilation was used, i.e., a lower tidal volume and slightly higher PEEP levels [55, 56]. However, other studies have failed to show significant changes after 2010 [57, 58]. Thus, it is unlikely that the metabolic changes reported in the present study are due to different mechanical ventilation strategies in the two groups. Fourth, ARDS was diagnosed according to the AECC, followed when the first cohort was recruited.

## Conclusions

In summary, we have characterized a specific metabolomic fingerprint that allows the discrimination between ARDS due to SARS-CoV-2 or to H1N1-2009 influenza virus in ARDS patients. The description of the metabolic alterations herein reported will help better understand the pathobiology of ARDS and its different causes and may have translational implications for biomarker discovery and the design of novel therapeutic targets.

## Supplementary Information

**Additional file 1.** **Table S1:** Metabolic Pathway analysis results. **Figure S1:** Boxplot figures of the metabolites found significant different (*p*-value < 0.05) between H1N1 influenza and COVID-19 patients.

## Data Availability

The datasets generated and/or analysed during the current study will be available in a online repository after acceptance.
