# Supplementary material for: Metabolomic diferences between COVID-19 and H1N1 influenza induced ARDS
Source: Crit Care. 2021 Nov 15;25:390. doi: 10.1186/s13054-021-03810-3 (PMC8591432; doi:10.1186/s13054-021-03810-3)

Table S1: Metabolic Pathway analysis results.

| Metabolic Pathway | P-value | False Discovery Rate | Impact |
| --- | --- | --- | --- |
| Propanoate metabolism | 0.0025153 | 0.056394 | 0.03846 |
| Aminoacyl-tRNA biosynthesis | 0.0046121 | 0.056394 | 0.3448 |
| Arginine and proline metabolism | 0.0049566 | 0.056394 | 0.1 |
| Pantothenate and CoA biosynthesis | 0.006445 | 0.056394 | 0.11112 |
| Valine, leucine and isoleucine biosynthesis | 0.015462 | 0.090193 | 0 |
| Valine, leucine and isoleucine degradation | 0.015462 | 0.090193 | 0.03774 |
| Ether lipid metabolism | 0.034605 | 0.13072 | 0 |
| Glycerophospholipid metabolism | 0.037064 | 0.13072 | 0.11538 |
| Ubiquinone and other terpenoid-quinone biosynthesis | 0.045297 | 0.13072 | 0.125 |
| Tyrosine metabolism | 0.045297 | 0.13072 | 0.11628 |
| Glycolysis / Gluconeogenesis | 0.04777 | 0.13072 | 0.08571 |
| Phenylalanine, tyrosine and tryptophan biosynthesis | 0.048555 | 0.13072 | 0.75 |
| Phenylalanine metabolism | 0.048555 | 0.13072 | 0.33333 |
| beta-Alanine metabolism | 0.057821 | 0.13492 | 0.04762 |
| Nicotinate and nicotinamide metabolism | 0.057821 | 0.13492 | 0.04762 |
| Glycerolipid metabolism | 0.074724 | 0.15333 | 0.09091 |
| Fatty acid degradation | 0.090493 | 0.15333 | 0.02041 |
| Histidine metabolism | 0.09462 | 0.15333 | 0.06667 |
| Galactose metabolism | 0.099266 | 0.15333 | 0 |
| Butanoate metabolism | 0.10068 | 0.15333 | 0.06667 |
| Porphyrin and chlorophyll metabolism | 0.10068 | 0.15333 | 0.03846 |
| Glutathione metabolism | 0.10068 | 0.15333 | 0.02703 |
| Alanine, aspartate and glutamate metabolism | 0.11078 | 0.15333 | 0.53125 |
| Glyoxylate and dicarboxylate metabolism | 0.11794 | 0.15333 | 0.11538 |
| D-Glutamine and D-glutamate metabolism | 0.11866 | 0.15333 | 0.5 |
| Nitrogen metabolism | 0.11866 | 0.15333 | 0.25 |
| Arginine biosynthesis | 0.12041 | 0.15333 | 0.25 |
| Pyruvate metabolism | 0.12267 | 0.15333 | 0.07408 |
| Glycine, serine and threonine metabolism | 0.17722 | 0.21389 | 0.02564 |
| Citrate cycle (TCA cycle) | 0.1972 | 0.23006 | 0.03448 |
| Pyrimidine metabolism | 0.23005 | 0.25162 | 0.01695 |
| Purine metabolism | 0.23005 | 0.25162 | 0.01163 |
| Selenocompound metabolism | 0.43203 | 0.45821 | 0 |
| Lysine degradation | 0.79059 | 0.79059 | 0.05 |
| Biotin metabolism | 0.79059 | 0.79059 | 0 |

Figure S1: Boxplot figures of the metabolites found significant different (p-value<0.05) between H1N1 influenza and COVID-19 patients.


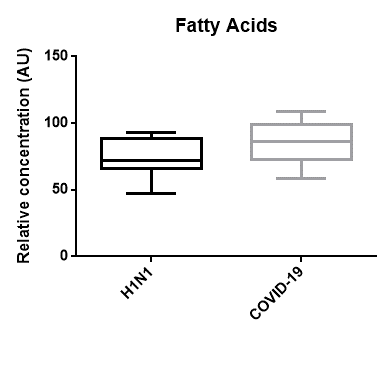

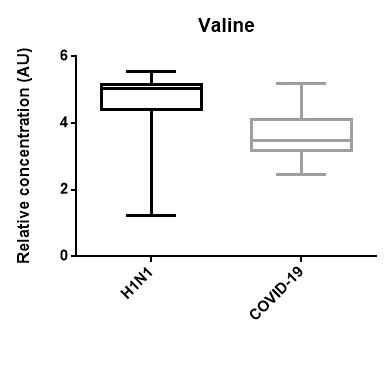


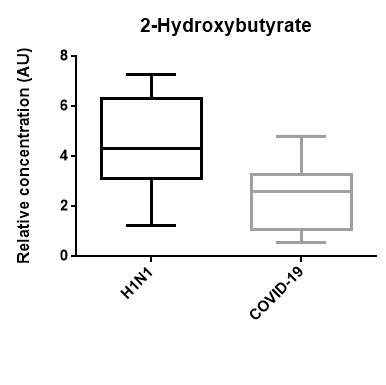

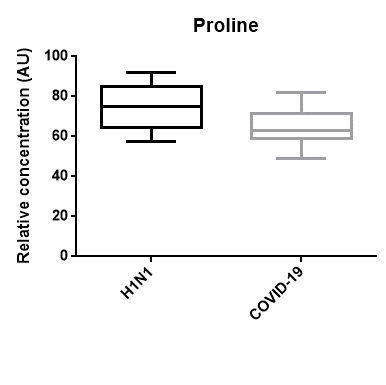


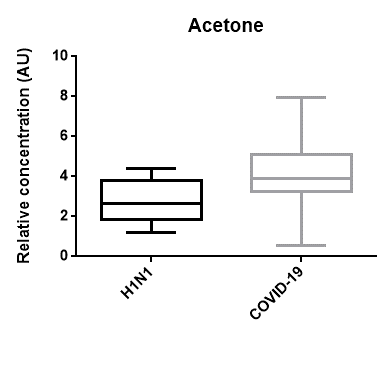

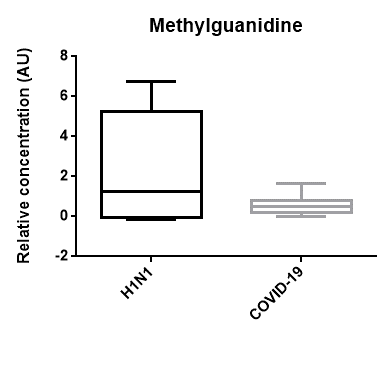


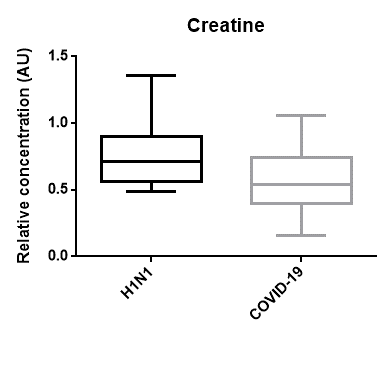

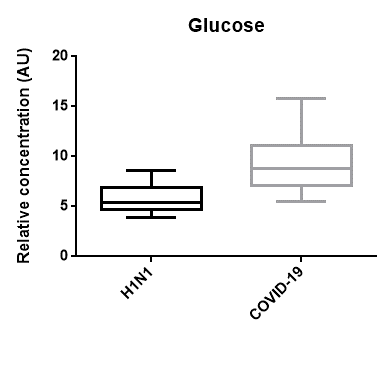


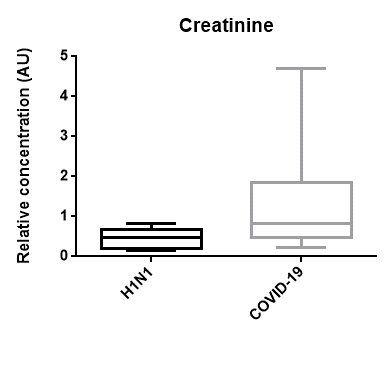

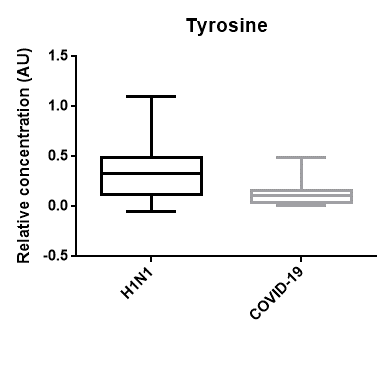

Supplement: Supplementary file 1 — Additional file 1. Table S1: Metabolic Pathway analysis results. Figure S1: Boxplot figures of the metabolites found significant different (p-value < 0.05) between H1N1 influenza and COVID-19 patients. [file 13054_2021_3810_MOESM1_ESM.docx]
